# Protective mechanism of rhubarb anthraquinone glycosides in rats with cerebral ischaemia–reperfusion injury: interactions between medicine and intestinal flora

**DOI:** 10.1186/s13020-020-00341-x

**Published:** 2020-06-05

**Authors:** Qiuying Li, Ying Guo, Xiahui Yu, Wenhong Liu, Liping Zhou

**Affiliations:** 1 College of Life Science, Zhejiang Chinese Medical University, Hangzhou, 310053 China; 2 College of Basic Medicine, Zhejiang Chinese Medical University, Hangzhou, 310053 China

**Keywords:** Rhubarb, Anthraquinone glycosides, Intestinal flora, Metabolism in vitro and in vivo, Cerebral ischaemic injury, The brain-gut axis

## Abstract

**Background:**

Anthraquinone glycosides extracted from rhubarb have been proven to have significant therapeutic effects on ischaemic stroke. It is well known that anthraquinone glycosides are not easily absorb. Thus, how can rhubarb anthraquinone glycosides (RAGs) exert protective effects on the brain? Is this protective effect related to interactions between RAGs and intestinal flora?

**Methods:**

The model used in this study was established by middle cerebral artery occlusion (MCAO) and reperfusion. Twenty-seven adult male Sprague–Dawley (SD) rats were randomly divided into 3 groups: the normal group (A) (non-MCAO + 0.5% sodium carboxymethyl cellulose (CMC-Na)), model group (B) (MCAO + 0.5% CMC-Na) and medicine group (C) (MCAO + RAGs (15 mg/(kg day)). The rats were fed by gavage once a day for 7 days. Fresh faeces were collected from the normal group to prepare the intestinal flora incubation liquid. Add RAGs, detect the RAGs and the corresponding anthraquinone aglycones by HPLC–UV at different time points. On the 8th day, the rats were euthanized, and the colonic contents were collected and analysed by high-throughput sequencing. In addition, 12 adult male SD rats were randomly divided into 2 groups: the normal group (D) (non-MCAO + RAGs (15 mg/(kg day)) and model group (E) (MCAO + RAGs (15 mg/(kg day)). The rats were fed by gavage immediately after reperfusion. Blood was collected from the orbital venous plexus, and the RAGs and anthraquinone aglycones were detected by HPLC–UV.

**Results:**

The abundance and diversity of the intestinal flora in rats decreased after cerebral ischaemia**–**reperfusion injury (CIRI). RAGs could effectively improve the abundance of the intestinal flora. In addition, in vitro metabolism studies showed that RAGs were converted into anthraquinone aglycones by intestinal flora. In the in vivo metabolism studies, RAGs could not be detected in the plasma; in contrast, the corresponding anthraquinone aglycones could be detected. Absorption of RAGs may be inhibited in rats with CIRI.

**Conclusions:**

CIRI may lead to intestinal flora disorder in rats, and after the administration of RAGs, the abundance of intestinal flora can be improved. RAGs can be metabolized into their corresponding anthraquinone aglycones by intestinal flora so that they can be absorbed into the blood.

## Background

According to the World Health Organization, cerebral ischaemia is the second leading cause of death and the third leading cause of disability in the world [1]. Cerebral ischaemia is caused by middle cerebral artery occlusion, which leads to some brain tissue damage, accompanied by inflammation and immune response [2]. According to the theory of TCM, cerebral ischaemia, which is also called stroke, can be induced by hyperactivity of liver Yang, disordered diet, stagnation of blood, etc. Among the causes, the main cause is blood stasis [3]. Prescriptions are commonly used to improve blood stasis in order to treat stroke, and these prescriptions include Xiexin Decoction (recorded in the *Synopsis of the Golden Chamber*), Didang Decoction (recorded in the *Treatise on Febrile Diseases*) and Dahuang Zhechong pill (recorded in the *Synopsis of the Golden Chamber*), all of which use rhubarb as the main ingredient.

Rhubarb, a species from *Polygonaceae* in the genus *Rheum*, is identified as the root and rhizome of *Rheum palmatum L.* in the *Chinese pharmacopeia*. In TCM theory, rhubarb can calm liver fire, promote blood circulation, and reduce blood stasis [4, 5], which is the theoretical basis for its treatment of stroke. The main active components of rhubarb are RAGs and the corresponding free anthraquinone [6, 7].

However, RAGs are glucosides with water-soluble sugars, and they are not easily absorb in the intestine and have low bioavailability [8]. Many scholars have studied the in vivo course and mechanism of these components. However, due to the complexity of TCM ingredients, there may be interactions between multiple components in the body. In addition, the body is a complex system, and there may be complex interactions between drug components and the body [9]. Therefore, there is much speculation about the mechanism of these ingredients, which are difficult to absorb but are capable of producing pharmacological activity. Many scholars believe that there is an inseparable relationship between intestinal flora and drug interactions, which may promote drug metabolism [10]. Modern pharmacology suggests that RAGs have significant protective effects against cerebral ischaemia injury in rats [11]. Likewise, RAGs are difficult to absorb and easily remain in the gut [12]. How can RAGs exert protective effects during cerebral ischaemia? Are these effects related to the microbes in the intestine?

We suspect that, on the one hand, RAGs can be hydrolysed into their glycoside ligands by intestinal flora [13] and thus may be absorbed into the blood and distributed to the brain to protect against ischaemic brain injury. On the other hand, according to *Shennong Materia Medica*, rhubarb is a medicine that is commonly used to treat constipation clinically by single-use or in combination prescriptions, such as Dachengqi Decoction [14]. In some cases, constipation may be regarded as a gastrointestinal disorder, and the intestinal flora play a vital role in gut motility [15]. Medicines that treat constipation, such as rhubarb, might be related to intestinal flora. RAGs are the main ingredients in rhubarb that act as laxatives [16], indicating that RAGs can affect intestinal flora.

There are many studies about the relationship between the brain and intestine in the classical Chinese medicine literature treatise on *Treatise on febrile diseases* and *Canon of Medicine*; these ancient books think that the dysfunctional stool caused by gastrointestinal dryness and heat can lead to the changes in mental disorders [17]. This belief leads to the “brain-gut connection” theory in TCM. The previously observed efficacy of rhubarb is relevant to the treatment of constipation [18] and stroke [19], and the classic rhubarb prescription Xiexin Decoction, which we mentioned before, is commonly used to treat stroke in TCM clinics through a purge. All of these findings are in line with the “Diseases in the brain should be treated from intestines” theory in TCM [20].

Modern pharmacology suggests that the bidirectional communication between the brain and the gastrointestinal tract, called the “brain-gut axis”, connects emotional and cognitive areas of the brain with gut functions [21]. This axis is a complex system, including the vagus nerve, sympathetic, endocrine, immune, humoural links and gut microbiota. Emerging evidence suggests that brain-gut interactions are significantly regulated by microbial populations [22, 23]. Many studies have found that intestinal flora play a key role in the occurrence and development of ischaemic stroke [24, 25]. Intestinal flora may participate in the pathogenesis of ischaemic stroke through chronic inflammation, the autonomic nervous system, and metabolism [26].

In our previous experiments [27], we found that RAGs can effectively reduce cerebral infarction size in rats with CIRI, and have a certain effect on intestinal flora. On this basis, inspired by the “brain-gut connection” theory, we speculated that the mechanism by which RAGs protect against CIRI was related to the intestinal flora. Therefore, we explored the interaction between RAGs and the intestinal flora in rats with cerebral ischaemia from two aspects. On the one hand, do RAGs have an effect on the intestinal flora in rats with cerebral ischaemia? This problem will be explored by high-throughput sequencing to determine the changes in the intestinal flora in rats with cerebral ischaemia before and after the administration of RAGs. On the other hand, how do RAGs, as glycosides that are difficult to absorb, enter the body to exert effects? Does the change in the intestinal flora affect the absorption and distribution of RAGs? This problem will be explored by determining the metabolic changes caused by RAGs in vitro and in vivo, combined with high-throughput sequencing results.

## Materials and methods

### Reagents and chemicals

LH-20 dextran gel was purchased from Shanghai Yuanye Biotechnology Ltd. (Shanghai, China). Standard substances of emodin-8-O-β-d-glucoside, chrysophanol-8-O-β-d-glucoside, rhein-8-O-β-d-glucoside, aloe-emodin-8-O-β-d-glucoside, physcion-8-O-β-d-glucoside, emodin, chrysophanol, rhein, aloe-emodin, and physcion were purchased from Nanjing Shizhou Biological Technology Ltd. (Nanjing, China). All the other reagents were chromatographically pure or analytically pure.

### Preparation and determination of anthraquinone glycoside extract

*Rheum palmatum* L. was presented by Traditional Chinese Herbal Pieces Ltd., Zhejiang Chinese Medical University (Zhejiang, China), and identified by Professor Chunchun Zhang of Zhejiang Chinese Medical University. The air-dried medicinal materials (0.5 kg) were crushed and passed through a 60-mesh sieve. The material was extracted by reflux extraction 3 times for 1 h each. The ratio of the material mass to the volume of 90% ethanol was 1:10, followed by filtration. The filtrate was concentrated by a rotary evaporator (RE 5298A, Shanghai, China).

The concentrate was extracted twice by dichloromethane and n-butanol at a volume ratio of 1:2. The n-butanol extract was collected and concentrated under vacuum pressure to obtain the dry powder. The powder was dispersed in water by ultrasound and then purified by LH-20 dextran gel. The eluent of 70% methanol was collected and concentrated to dry powder, which was used as the active constituent for the following experiments.

The total RAG content was determined by an ultraviolet–visible spectrophotometer (TU-1900, Beijing, China) with emodin-8-O-β-d-glucoside as the standard substance, and the content was 64.50%.

### Preparation of standard solution

Certain amounts of the emodin-8-O-β-d-glucoside, chrysophanol-8-O-β-d-glucoside, rhein-8-O-β-d-glucoside, aloe-emodin-8-O-β-d-glucoside, physcion-8-O-β-d-glucoside, emodin, chrysophanol, rhein, aloe-emodin, and physcion standards were dissolved in methanol to form a mixed reference solution. Further dilution was performed to yield a series of solutions for the establishment of the calibration curve, limit of detection (LOD), and limit of quantification (LOQ) for each compound. The peak area of the different concentrations of the mixed standard solution was determined by HPLC, and the standard curve was prepared with the peak area (*Y*) as the ordinate and the mixed standard solution sample size (*X*) as the abscissa.

### HPLC analysis

The experiments were performed using a LC-20A (SHIMADZU, Japan) High-Performance Liquid Chromatograph and a SHIMADZU ODS3-C_18_ chromatographic column (4.6 mm × 250 mm, 5 µm). The mobile phase consisted of acetonitrile as Solvent A and 1% formic acid as Solvent B at a flow rate of 1 mL/min. The column temperature was maintained at 30 °C, and the injection volume was 20 µL.

The gradient elution program of the standard solution included 35–55% A at 0–10 min; 55–75% A at 10–30 min; 75% A at 30–31 min. The detection wavelength was 254 nm.

The gradient elution program of the transformation assay in vitro included 20–50% A at 0–10 min and 50–90% A at 10–35 min. The detection wavelength was 280 nm.

The gradient elution program of the transformation assay in vivo included 35–55% A at 0–10 min; 55–75% A at 10–30 min; 75–95% A at 30–40 min. The detection wavelength was 254 nm.

### Animals and group

Thirty-nine adult male SD rats (8–10 weeks) weighing 280–300 g were purchased from the Zhejiang Chinese Medical University Laboratory Animal Research Center (certification: SCXK (Zhejiang) 2014-0001). All the animals were handled according to the animal protection committee of Zhejiang Chinese Medical University. The animals were kept under constant laboratory conditions (20–25 °C 60 ± 5% humidity) and a 12 h light/dark cycle. The rats were allowed free access to food and water. Twelve hours before surgery, only water was available.

Twenty-seven of the adult male SD rats were randomly divided into 3 groups: the normal group (A) (non-MCAO + 0.5% carboxymethylcellulose sodium (CMC-Na)), model group (B) (MCAO + 0.5% CMC-Na) and medicine group (C) (MCAO + RAGs (15 mg/(kg day)), and the medicine was administered by gavage immediately after reperfusion.

The other 12 adult male SD rats were randomly divided into 2 groups: the normal group (D) (non-MCAO + RAGs (15 mg/(kg day)) and model group (E) (MCAO + RAGs (15 mg/(kg day)), which was treated by gavage immediately after reperfusion.

### Establishment of the MCAO rat model and drug administration

The MCAO rat model was established as previously described [28]. In brief, after 12 h of fasting, the rats were anaesthetized by intraperitoneal injection of 2% pentobarbital sodium (0.2 mL/100 g, provided by Zhejiang Laboratory Animal Center). Subsequently, the right common carotid artery (CCA), external carotid artery (ECA), and internal carotid artery (ICA) were exposed through a neck midline incision, carefully separated from the vagus nerve and ligated, and the pterygopalatine artery of the ICA was identified. A monofilament nylon suture was inserted into the ICA through the ECA (approximately 18–20 mm from the bifurcation). One hour after MCAO, reperfusion was performed by removing the nylon suture.

After recovering from anaesthesia, the rats in groups A, B, and C were fed by gavage once a day for 7 days. The animals were allowed free access to food and water.

### PCR amplification of the V3-V4 region of the bacterial 16S rRNA gene and Illumina sequencing

On the 8th day after modelling, the rats in groups A, B and C were anaesthetized with 2% pentobarbital sodium (0.2 mL/100 g) and were sacrificed. The colonic contents were collected aseptically. Five colonic content samples were selected at random from each group (A, B, and C) for PCR amplification of the V3-V4 region of the bacterial 16S rRNA gene and Illumina sequencing. The DNA was extracted by the E.Z.N. A Soil DNA Kit (OMEGA). The bacterial genomic DNA was amplified with the forward primer GACTACHVGGGTATCTAATCC and the reverse primer CCTACGGGNGGCWGCAG. The sequencing was performed on an Illumina MiSeq Benchtop Sequencer provided by Genesky Biotechnologies Inc., Shanghai, China. The merged paired-end reads from the DNA fragments were analysed by next-generation sequencing FLASH. The sequencing reads were based on the unique barcode and were analysed by the QIIME software package (Quantitative Insights Into Microbial Ecology) and UPARSE pipeline. In brief, the reads were filtered by QIIME quality filters (using the default setting for Illumina), and operational taxonomic units (OTUs) were picked using the UPARSE pipeline.

### Transformation assay in vitro

Fresh faeces of the rats in group A were collected, and sterilized normal saline was added at a mass volume ratio of 1:4 to prepare a suspension. The suspension was centrifuged at 2000 r/min for 10 min. The supernatant was transferred to a glass culture flask, TSB culture solution was added at a volume ratio of 1:9, and the bacteria were cultured in an anaerobic incubator for 12 h. The bacterial culture solution was collected and centrifuged at 4000 r/min for 15 min. The residue was collected and dissolved in 1 mL sterilized normal saline to obtain the intestinal flora solution of rats. One millilitre of RAGs solution (10 mg/mL) was added to 9 mL of TSB culture medium, and 50 μL of rat intestinal flora solution was added. In addition, the blank group (1 mL distilled water + 9 mL TSB culture medium + 50 μL intestinal flora solution) and the control group (1 mL drug solution + 9 mL TSB culture medium + 50 μL sterilized normal saline) were established. Each group was placed in a 37 °C anaerobic incubator and cultured for 0, 2, 4, 8, 12, 24 and 48 h. Then, 0.5 mL methanol was added to terminate the reaction, and the solution was vortexed for 2 min and centrifuged at 12,000 r/min for 10 min. Then, 0.7 mL supernatant was collected and dried by N_2_ flow. The residue was dissolved with 500 μL methanol and centrifuged for 10 min at 12,000 r/min, and 20 μL supernatant was collected for analysis.

### Transformation assay in vivo

Blood was collected from the orbital venous plexus of the rats in groups D and E at 0, 0.083, 0.25, 0.5, 0.75, 1, 2, 4, 6, 8, and 12 h after administration, using 3.8% sodium citrate as the anticoagulant. After centrifugation at 4000 r/min for 10 min, 100 μL plasma was collected, 300 μL methanol was added, and the solution was vortexed for 5 min and centrifuged for 15 min at 12,000 r/min. A total of 300 μL of supernatant was dried by N_2_ flow. The residue was redissolved with 300 μL methanol, vortexed for 2 min, centrifuged for 10 min at 12,000 r/min, and the supernatant was collected for analysis.

### Statistics analysis

All the statistical analyses were performed using SPSS 17.0 software. All the data are presented as the mean ± SEM. The statistical significance of the study was analysed by one-way ANOVA and Tukey’s test. The statistical significance level was set at *p*  <  0.05.

## Results

### Calibration equations

The regression equation of emodin-8-O-β-d-glucoside was *Y *= 14.946*X *+ 0.216, R^2^ = 0.9996, the equation of chrysophanol-8-O-β-d-glucoside was *Y *= 49.099*X *+ 0.175, R^2^ = 0.9994, the equation of rhein-8-O-β-d-glucoside was *Y *= 36.850*X *+ 0.339, R^2^ = 0.9992, the equation of aloe-emodin-8-O-β-d-glucoside was *Y *= 34.852*X *+ 0.490, R^2^ = 0.9994, the equation of physcion-8-O-β-d-glucoside was *Y *= 21.556*X *+ 0.184, R^2^ = 0.9996, the equation of emodin was *Y *= 55904*X − *8753, R^2^ = 0.9993, the equation of chrysophanol was *Y *= 27829*X *+ 201.5, R^2^ = 0.9990, the equation of rhein was *Y *= 42231*X *+ 25,290, R^2^ = 0.9998, the equation of aloe-emodin was *Y *= 86620*X *+ 2508.5, R^2^ = 0.9997, and the equation of physcion was *Y *= 39479*X *+ 58,548, R^2^ = 0.9990. These results indicate that the ten anthraquinones had a good linear relationship in the concentration range of 1.086–34.750 μg/mL, 0.430–13.750 μg/mL, 0.794–25.413 μg/mL, 0.840–26.875 μg/mL, 0.521–16.669 μg/mL, 0.262–16.750 μg/mL, 0.398–12.750 μg/mL, 0.477–15.250 μg/mL, 0.227–14.500 μg/mL, and 0.186–23.750 μg/mL, respectively.

### Anthraquinone glycosides content

**Fig. 1:**
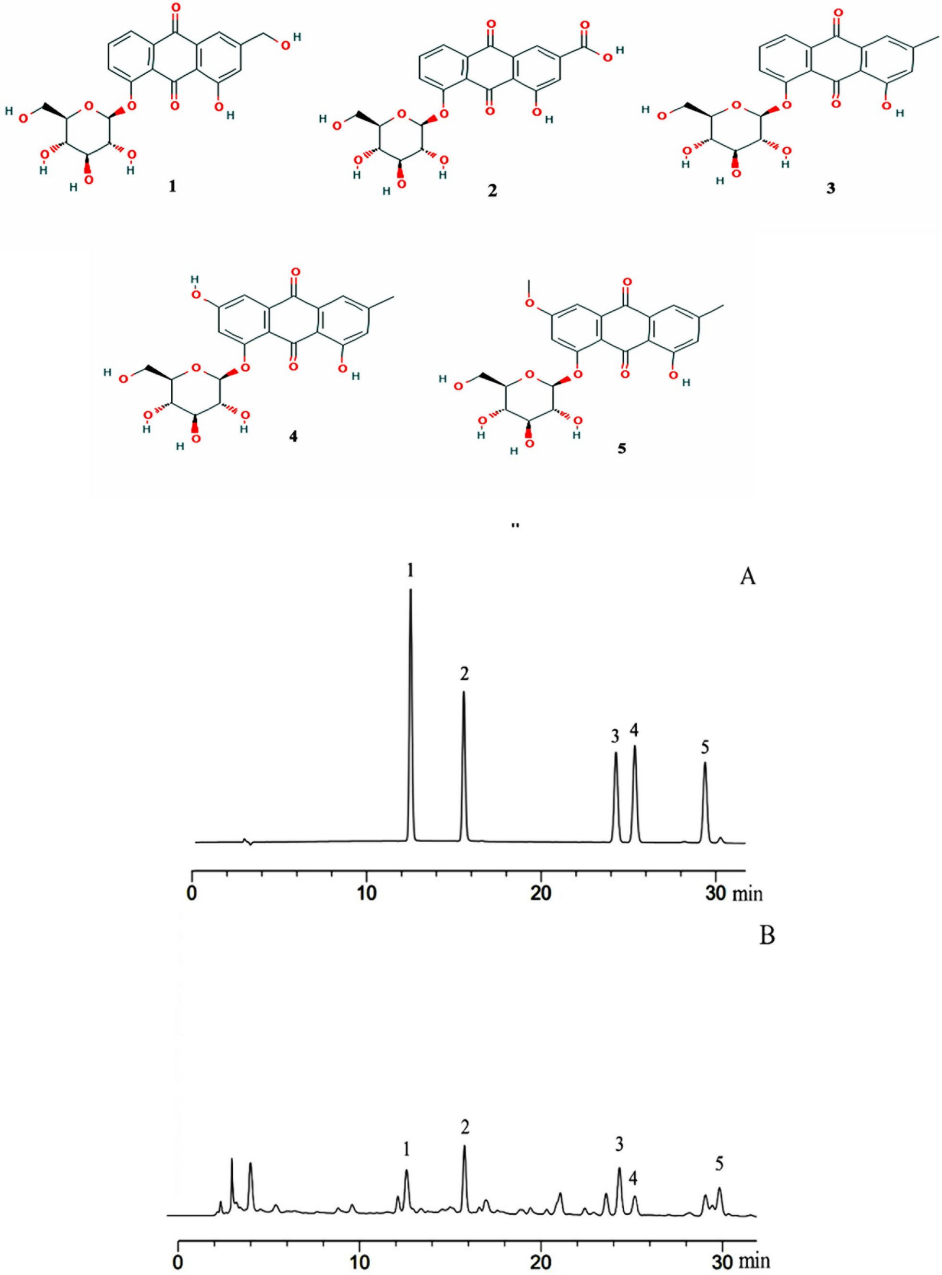
The structure of aloe-emodin-8-O-β-d-glucoside (1), rhein-8-O-β-d-glucoside (2), chrysophanol-8-O-β-d-glucoside (3), emodin-8-O-β-d-glucoside (4), and physcion-8-O-β-d-glucoside (5), the HPLC chromatogram of the mixed reference substance **a** and purified anthraquinone glycosides from *Rheum palmatum* L. **b**

The contents of emodin-8-O-β-d-glucoside, chrysophanol-8-O-β-d-glucoside, rhein-8-O-β-d-glucoside, aloe-emodin-8-O-β-d-glucoside, and physcion-8-O-β-d-glucoside are 5.55%, 4.23%, 6.91%, 6.37%, and 2.00%, respectively (Fig. 1). These five anthraquinone glycosides account for more than 25% of the total anthraquinone glycosides.

### RAGs increased the abundance of intestinal flora in rats with CIRI

**Fig. 2:**
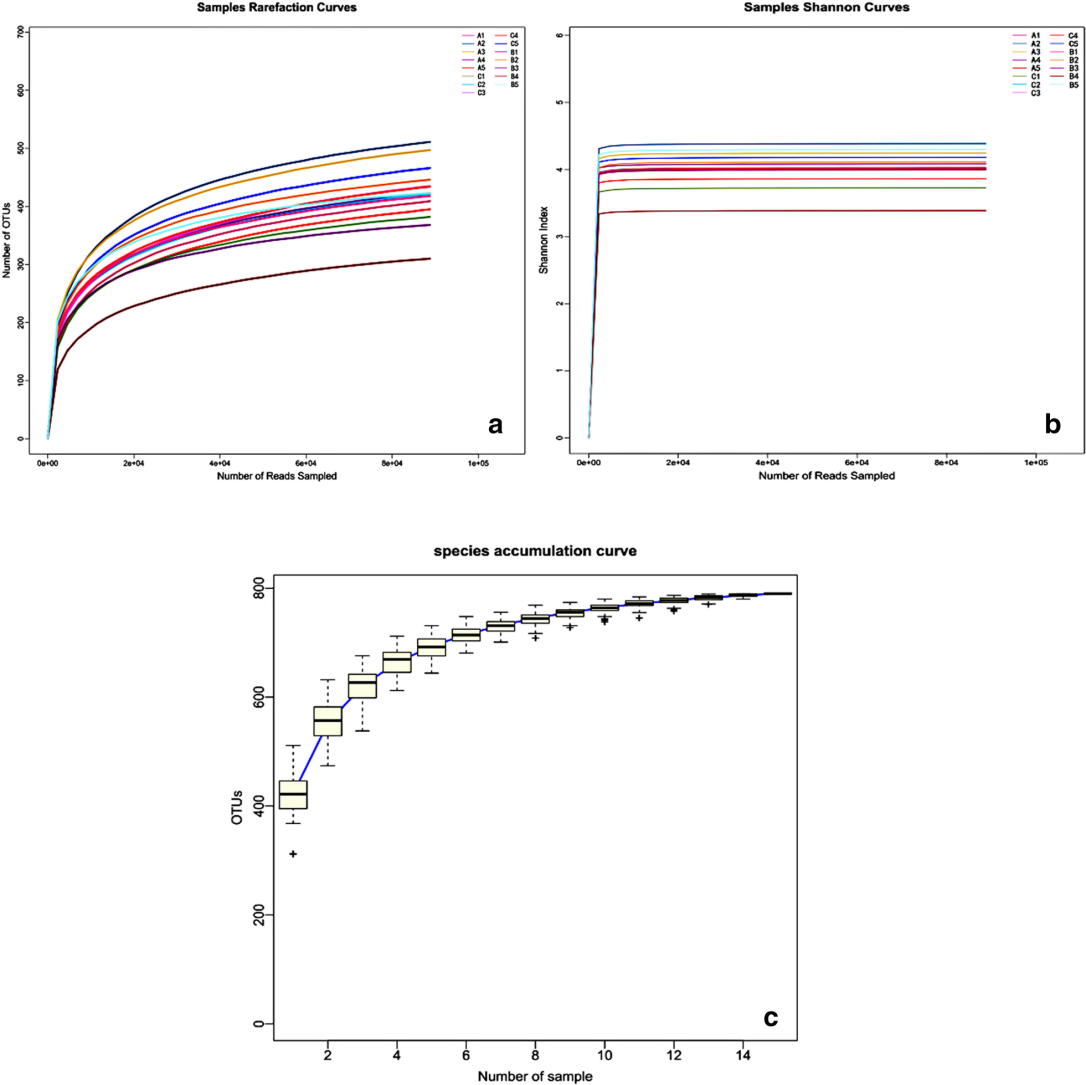
Rationality analysis of the sample sampling amount. **a** Rarefaction analysis of the different samples, **b** Shannon–Wiener analysis of the different samples, **c** Species accumulation analysis of the different samples

To assess the effect of RAGs on intestinal flora in rats with CIRI, we analysis the colonic contents in different groups by high-throughput sequencing. All the rarefaction curves (99% similarity of OTUs) of the 3 groups plateaued (Fig. 2a), which meant that the majority of the sequences were involved in the analysis process. However, the OTU density was larger in the upper layer than in the lower layers. The same trend was found in the species accumulation curves (Fig. 2c) and Shannon–Wiener curves (Fig. 2b), which approaches a saturation plateau, indicating that the database of the 16S rRNA gene sequences was very abundant and reflected the vast majority of microbial information.

**Fig. 3:**
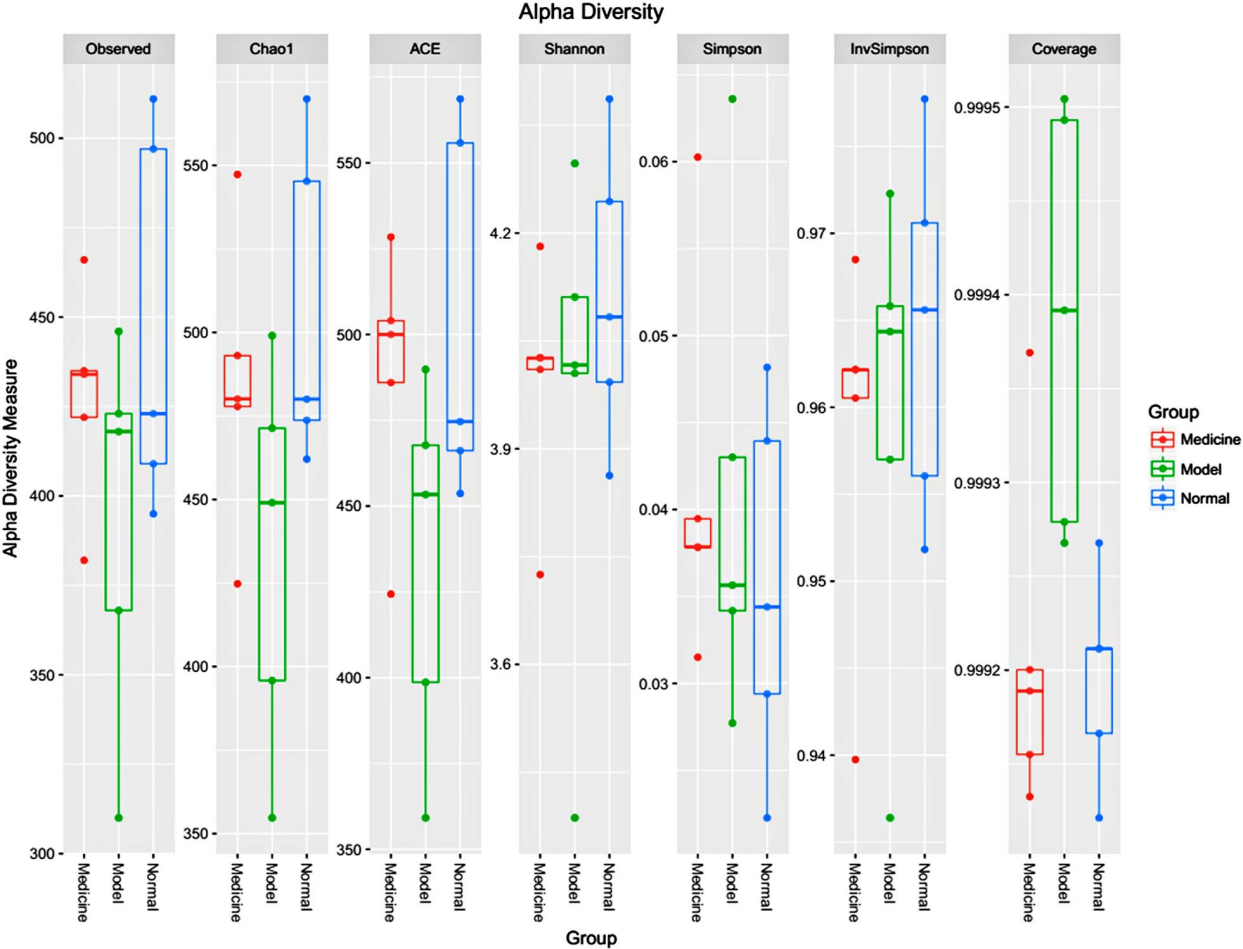
Abundance and diversity indexes are relative to each sample (OTU cut-off of 0.03)

We compared the microbial richness of groups A, B, and C, as estimated by the Chao1 and ACE indexes, and assessed the biodiversity by the anon parametric Shannon index and Simpson index. In our calculations, we took into account the OTU distance unit cut-off of 0.03 (Fig. 3). We found that the intestinal flora abundance of the normal group (A) was the highest, followed by the medicine group (C) and the model group (B). Additionally, from the perspective of intestinal flora biodiversity, the normal group (A) was the highest, and the medicine group (C) was the lowest. All of these results showed that intestinal flora abundance and diversity are decreased after CIRI. After the administration of RAGs, the abundance of the intestinal flora increased, but the diversity decreased.

### RAGs improved the structure of intestinal flora in rats with CIRI

The fifteen samples comprised different numbers of OTUs and OTU abundances. All the sequences were classified from phylum to genus according to the program default setting. The sequences that could not be classified into any known group were assigned as NO-Rank. These microorganism OTUs can be assigned to 14 different phyla, 45 families or 90 genera.

**Fig. 4:**
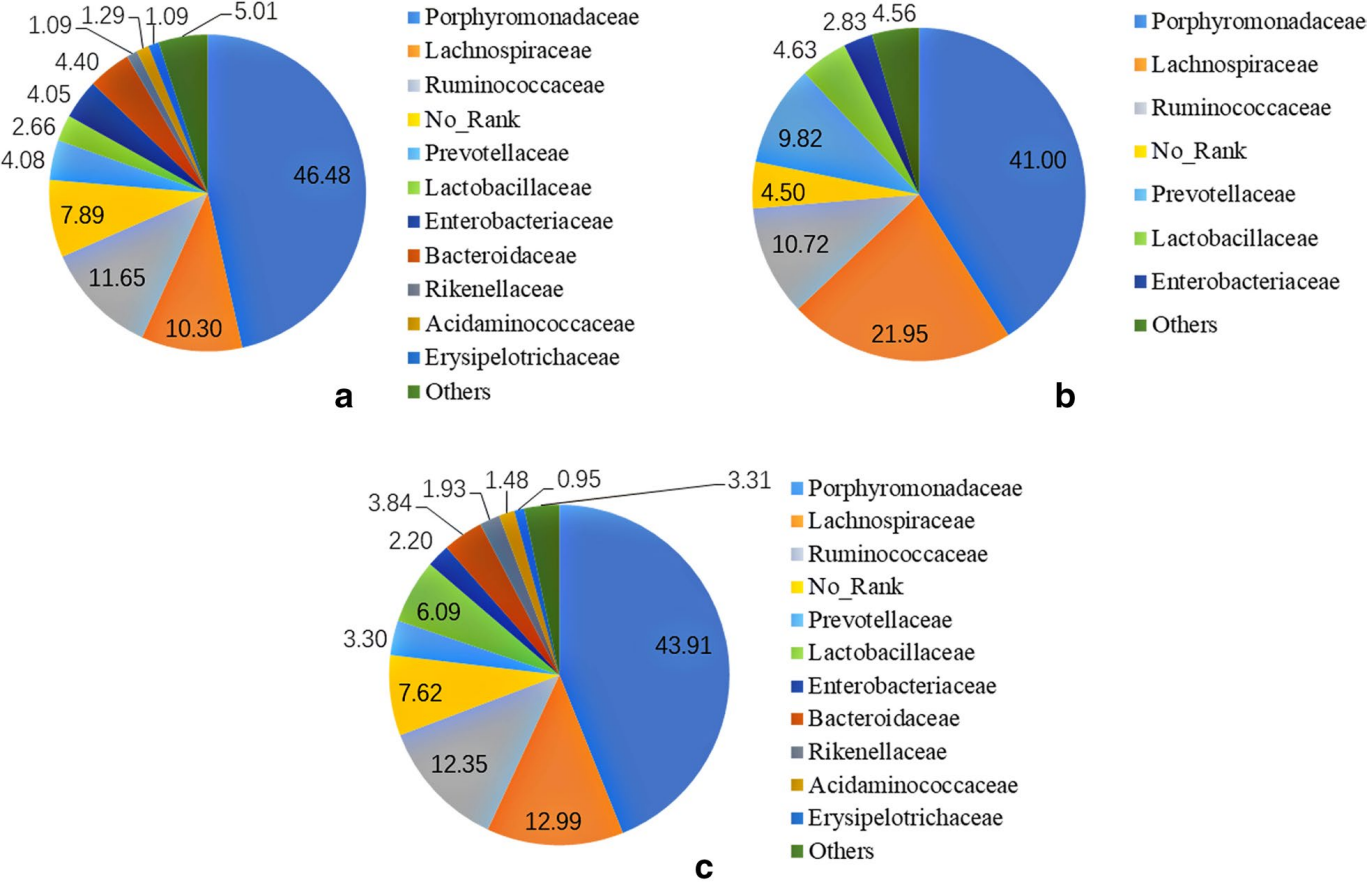
Relative abundance (> 1%) of the intestinal flora at the family level in different groups (percent). **a** The mean abundance of the intestinal flora at the family level in the normal group. **b** The mean abundance of the intestinal flora at the family level in the model group. **c** The mean abundance of the intestinal flora at the family level in the medicine group

At the family level, there were 6 shared families (abundance > 1%) among the 45 families that existed in all the samples (Fig. 4); these families included *Porphyromonadaceae*, *Lachnospiraceae*, *Ruminococcaceae, Prevotellaceae*, *Lactobacillaceae*, and *Enterobacteriaceae*. The most abundant family in groups A, B, and C was *Porphyromonadaceae*, which accounted for 46.48%, 41.00%, and 43.91%, respectively. The relative abundance of *Lachnospiraceae* and *Prevotellaceae* in group B was significantly higher than that in groups A and C. In addition, it was obvious that the intestinal flora of groups B and C had high similarity at the family level (Fig. 3). This observation suggests that the relative abundance of *Lachnospiraceae* and *Prevotellaceae* was increased after CIRI, and the diversity of dominant bacteria in the first grade was reduced. After the administration of RAGs, the relative abundance of *Lachnospiraceae* and *Prevotellaceae* decreased significantly, and the balance of dominant bacteria was improved.

**Fig. 5:**
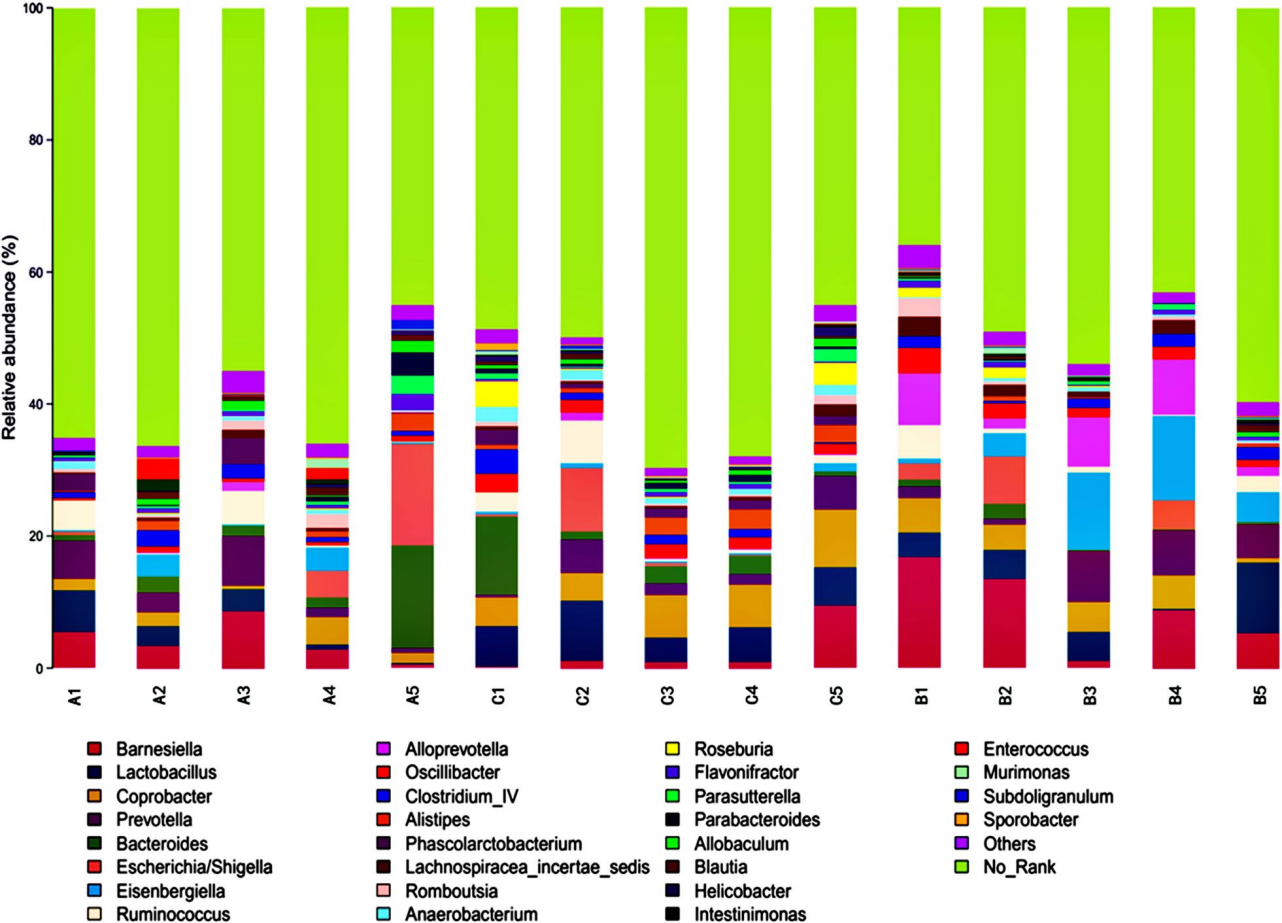
Bacterial composition of the different communities at the genus level. The relative abundance of the different bacterial genera within the different communities. The sequences that could not be classified into any known group were assigned as No Rank

The detected OTUs were distributed among 90 different bacterial genera. NO-Rank was the most abundant division (Fig. 5), and the mean OTU abundance in groups A, B and C was 59.45%, 48.34%, and 58.11%, respectively. The second-most abundant divisions in groups A, B and C were *Bacteroides* (4.40%),*Barnesiella* (9.15%) and *Coprobacter* (6.39%), respectively. Combined with the data of Fig. 4, *Alloprevotella* and *Lachnospiracea*-*incertae*-*sedis* are the genera specific to the group B samples; *Bacteroides*, *Alistipes,* and *Phascolarctobacterium* are common genera of groups A and C, but they are difficult to find in group B. Thus, specific bacteria may be affected after cerebral ischaemia–reperfusion in rats. After the administration of RAGs, these specific bacteria could be regulated so that the structure of intestinal flora can be restored to closely resemble that of normal rats.

### RAGs could effectively improve the imbalance of intestinal flora caused by CIRI

**Fig. 6:**
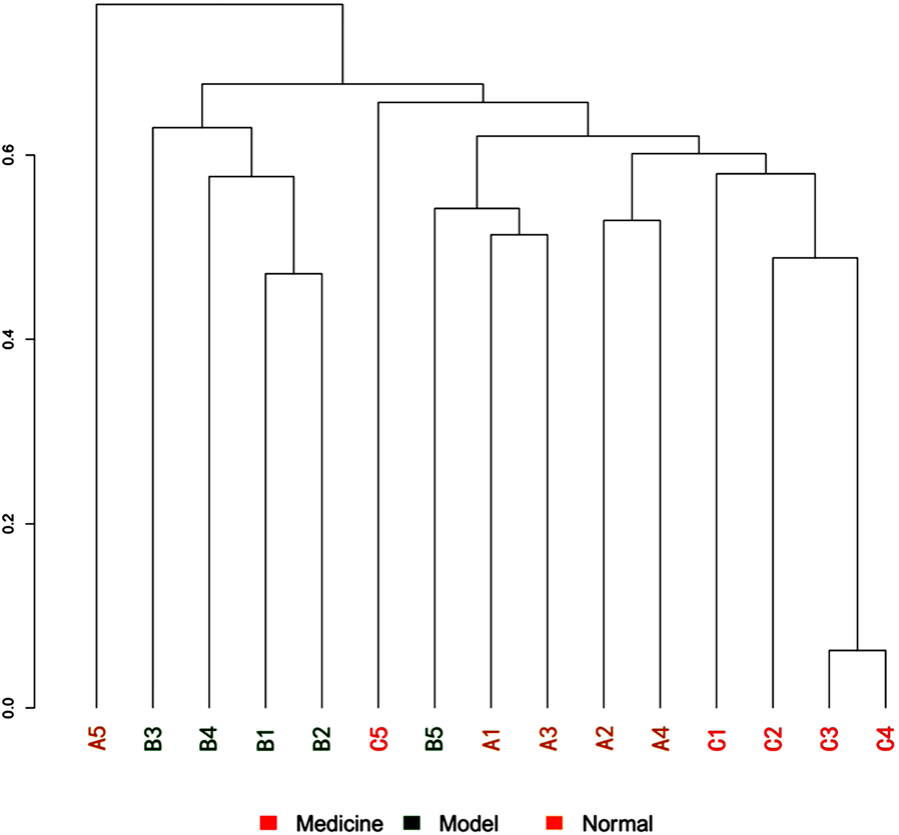
Multiple sample similarity trees

The multiple sample similarity trees were built based on the hierarchical clustering method by R software (Fig. 6). The intestinal bacteria of groups A and C were clustered together and then clustered with group B, indicating that most bacteria in groups A and C had a relatively close genetic relationship.

**Fig. 7:**
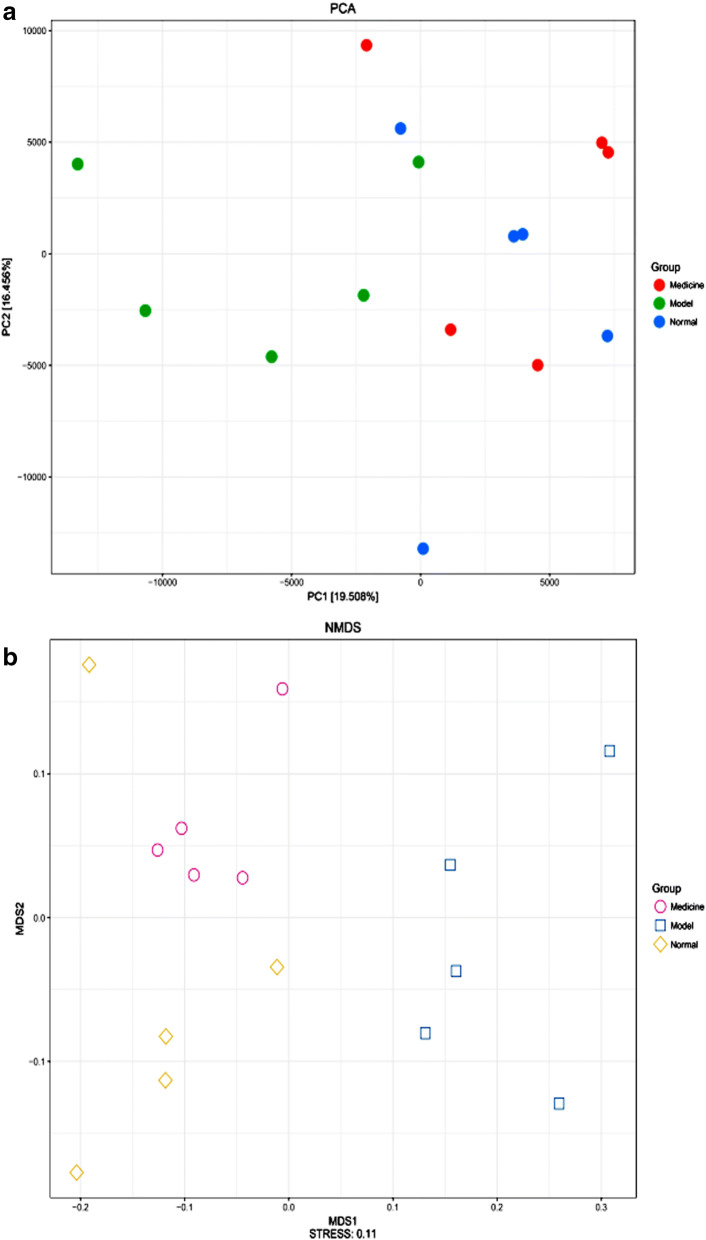
Sample sorting analysis. Scatter plot of PCA score showing similarity of the 15 bacterial communities based on UniFrac distance. Principal components (PCs) 1 and 2 explained 19.508% and 16.456% of the variance, respectively. NMDs showing the difference in bacterial communities according to Jaccard distance

The principal component analysis (PCA) score plot revealed that the intestinal bacteria of group B are harboured characteristic bacterial communities. The intestinal bacteria of groups A and C were closely related (Fig. 7a). The NMDS analysis based on the Jaccard distance also confirmed that the bacterial communities in group B were more similar than those in groups A and C (Fig. 7b).

**Fig. 8:**
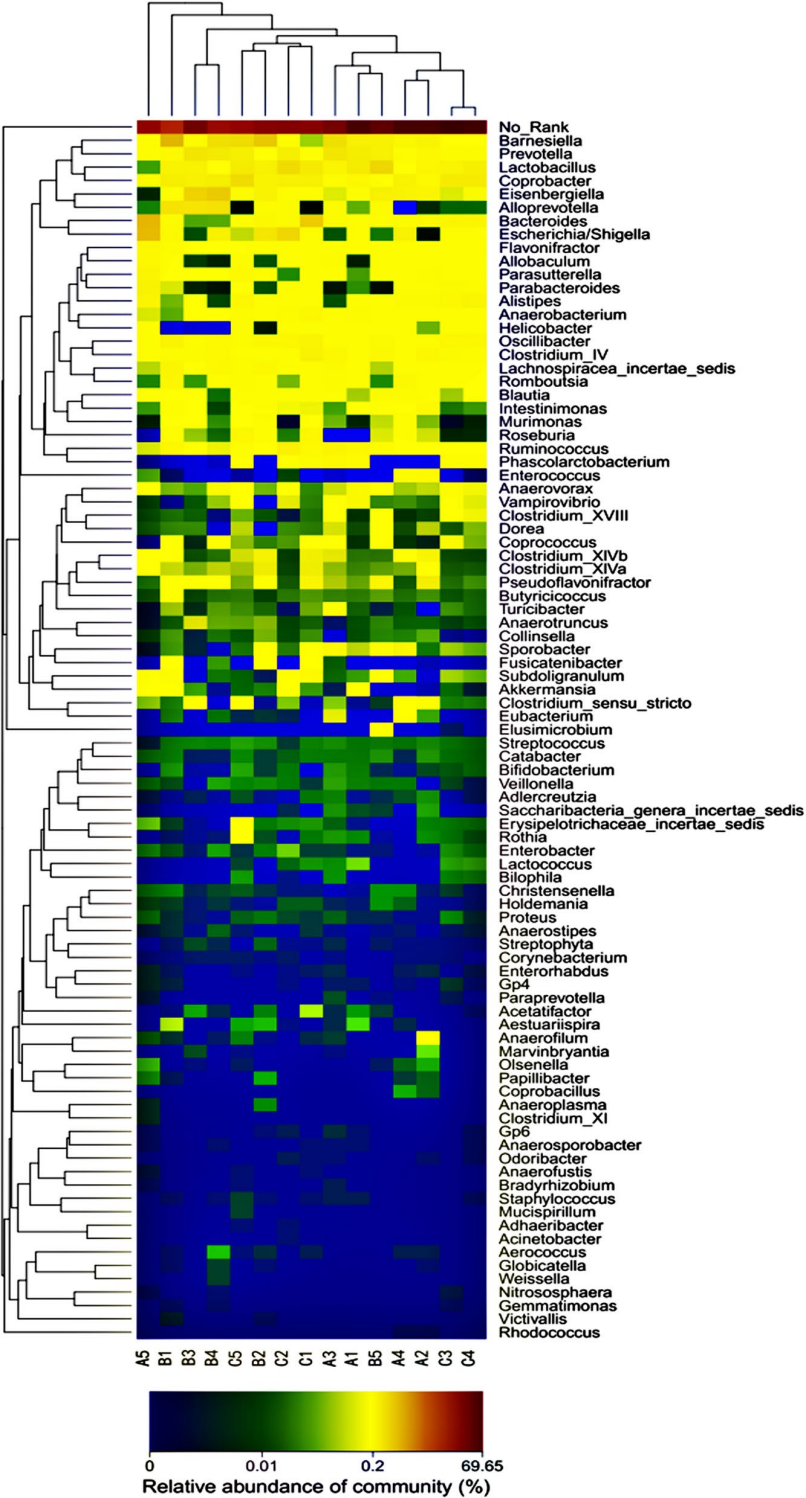
Bacterial distribution of the top 100 abundant genera among fifteen samples. The double hierarchical dendrogram shows the bacterial distribution. The heat map plot depicts the relative percentage of each bacterial genus within each sample. The colour in the figure represents the abundance of the species, and the colour from blue to red indicates that the abundance of the species is small to large

The genus and the relative abundances of intestinal bacteria were clustered as a heatmap, where darker colours indicate a higher abundance of the bacterial flora (Fig. 8). According to the heatmap, the highest similarity of the communities could be found in the samples from group A, and relatively high similarity was also found in groups B and C. These results indicate that RAGs could effectively improve the changes in intestinal flora caused by CIRI and improve the structure of intestinal flora, causing these parameters to return to normal.

### Intestinal flora could transform RAGs into anthraquinone aglycones in vitro

**Fig. 9:**
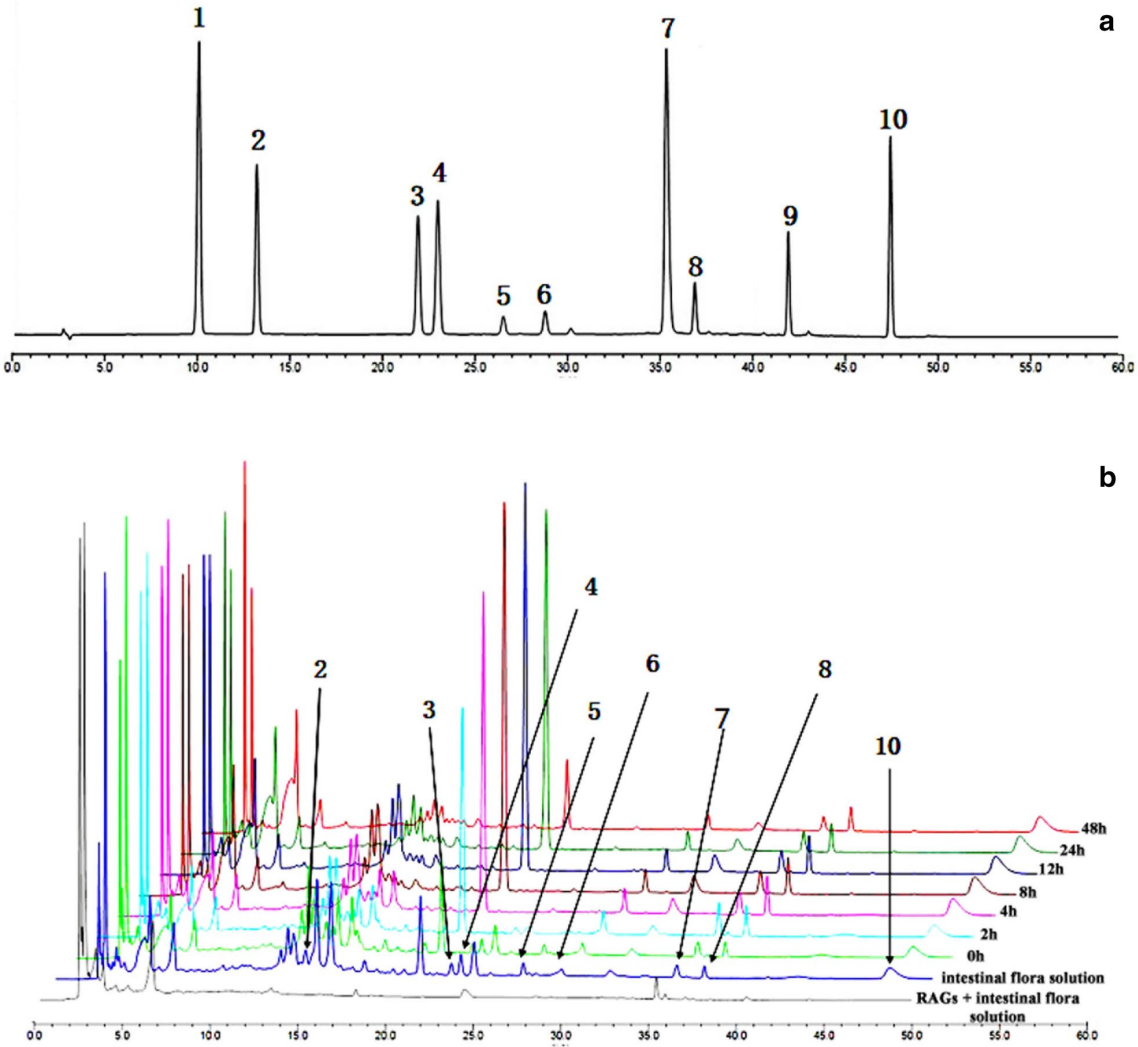
HPLC chromatogram of the mixed anthraquinone reference substance **a**, HPLC chromatogram of the changes in the RAGs at different time points of in vitro metabolism **b**. Aloe-emodin-8-O-β-d-glucoside (1), rhein-8-O-β-d-glucoside (2), chrysophanol-8-O-β-d-glucoside (3), emodin-8-O-β-d-glucoside (4), physcion-8-O-β-d-glucoside (5), aloe-emodin (6), rhein (7), chrysophanol (8), emodin (9) and physcion (10)

RAGs are not easily absorb in the intestine and its efficacy in vivo may be affected by the intestinal flora. We next determined whether the transformation of RAGs in vitro and in vivo is related to intestinal flora. The concentrations of rhein-8-O-β-d-glucoside, chrysophanol-8-O-β-d-glycosidase, emodin-8-O-β-d-glycosidase, and physcion-8-O-β-d-glycosidase were gradually reduced within 0–2 h. In addition, the concentrations of the corresponding anthraquinone aglycones increased gradually, indicating that the main pathway of RAGs metabolism in vitro was hydrolysis. After incubation for 8 h, the concentrations of free anthraquinone gradually decreased, indicating that the anthraquinone aglycones began to be decomposed by the intestinal flora and may further produce other metabolites (Fig. 9).

### RAGs could not be directly absorbed into the blood and need to be metabolized by intestinal flora first

**Fig. 10:**
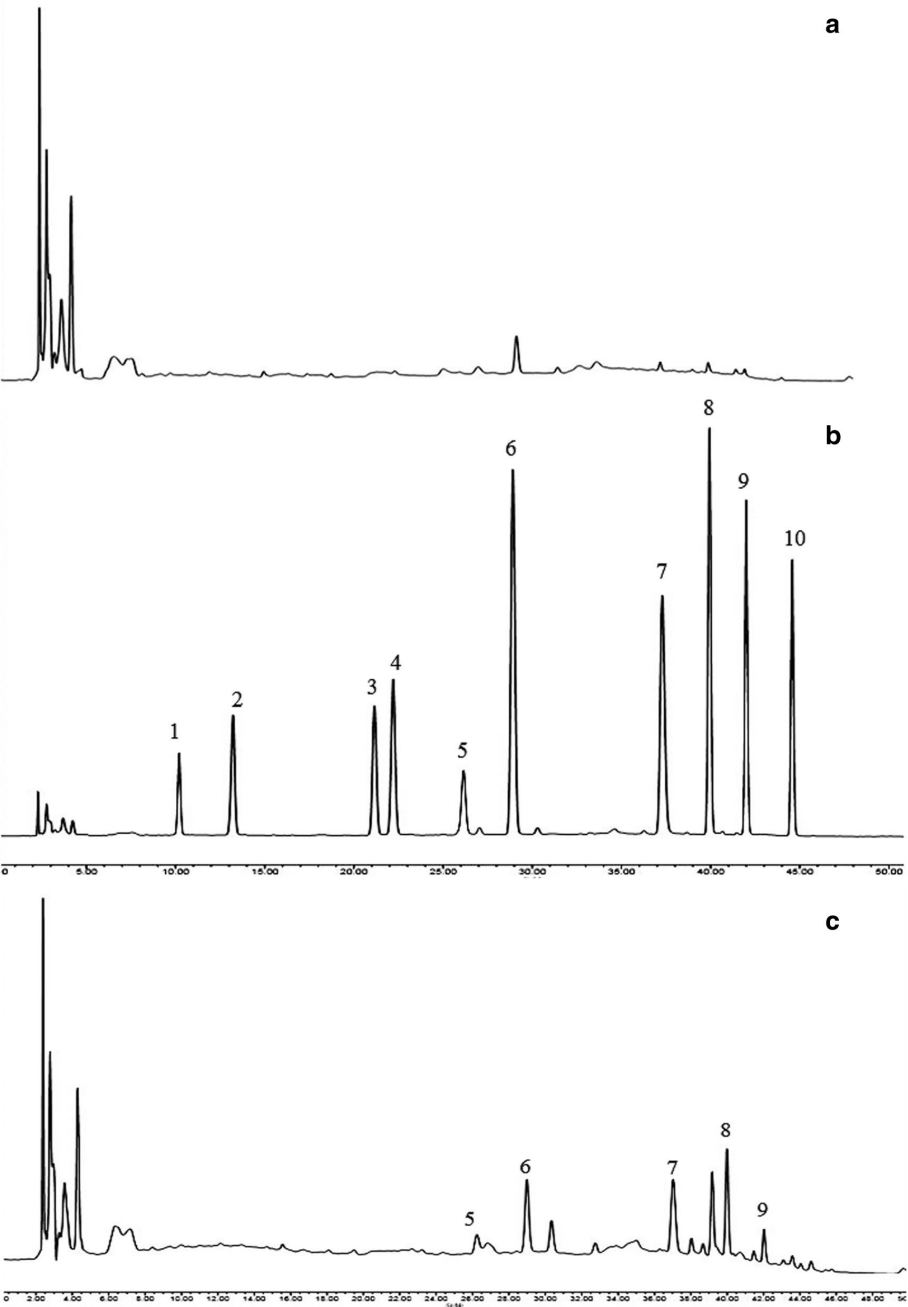
HPLC chromatogram of blank plasma **a**; HPLC chromatogram of mixed anthraquinone reference substance + blank plasma **b**; HPLC chromatogram of medicated plasma **c**, aloe-emodin-8-O-β-d-glucoside (1), rhein-8-O-β-d-glucoside (2), chrysophanol-8-O-β-d-glucoside (3), emodin-8-O-β-d-glucoside (4), physcion-8-O-β-d-glucoside (5), aloe-emodin (6), rhein (7), chrysophanol (8), emodin (9), and physcion (10)

The five anthraquinone glycosides components could not be detected in the plasma except for physcion-8-O-β-d-glucoside, while their corresponding anthraquinone aglycones could be detected (Fig. 10c). Combined with the tranformation results in vitro, it is suggested that RAGs can not be directly absorb into the blood and may be metabolized into anthraquinone aglycones by intestinal flora and then absorb in vivo.

**Fig. 11:**
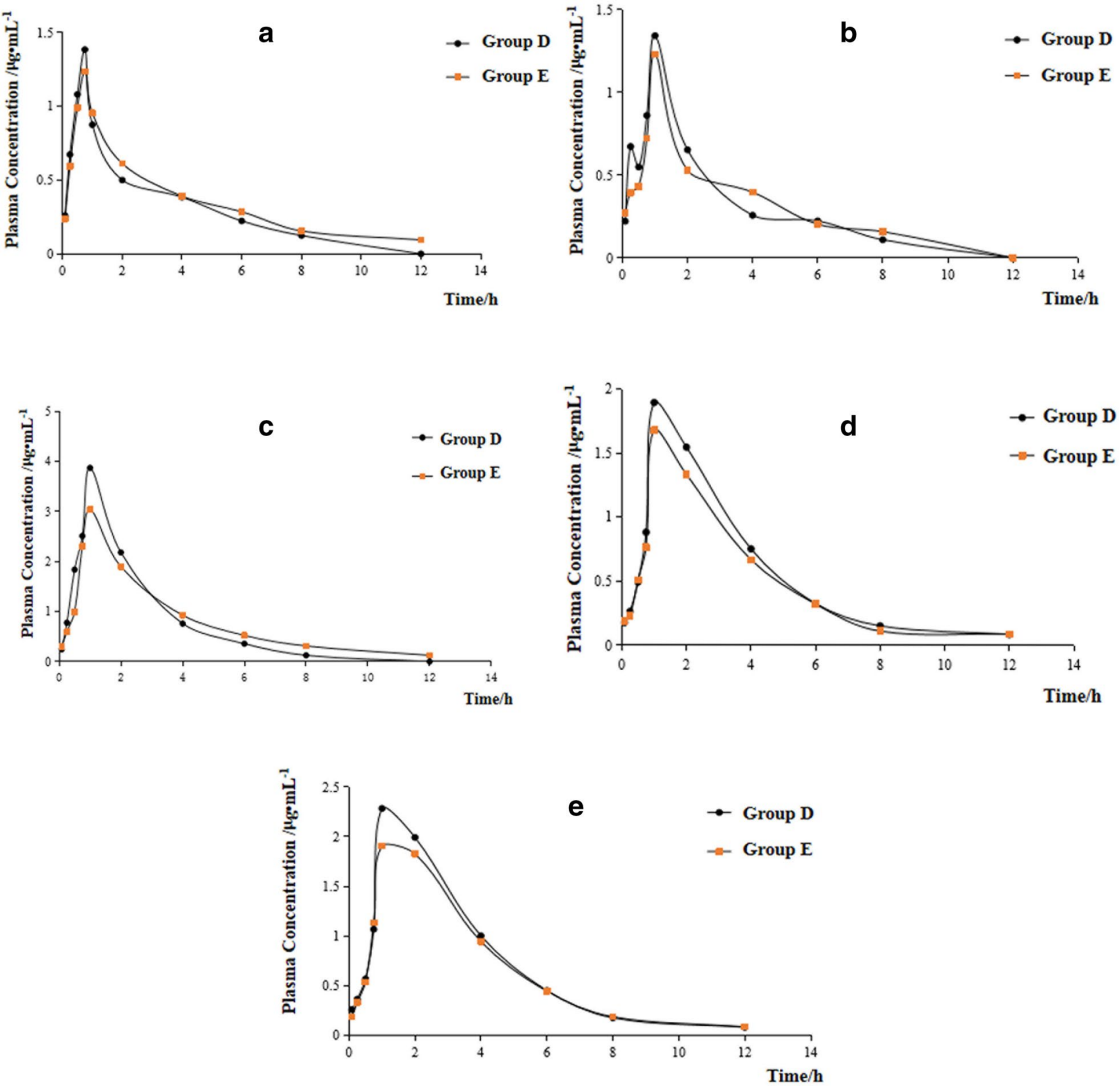
The metabolic process of anthraquinone compounds in rhubarb of rats in each group. Physcion-8-O-β-d-glucoside **a**, aloe-emodin **b**, rhein **c**, emodin **d**, chrysophanol **e**

The blood concentration peak time of physcion-8-O-β-d-glucoside was 0.75 h, and that of aloe-emodin, rhein, emodin, and chrysophanol was 1 h. The maximum concentrations of the 5 anthraquinones compounds in group D were higher than those in group E. The time when anthraquinones compounds in group D could not be detected was generally earlier than that in group E (Fig. 11), indicating that CIRI may inhibit the absorption of anthraquinones compounds.

## Discussion

The main findings of this study are as follows: (1) CIRI can reduce the abundance and diversity of the intestinal flora, and the structure of the intestinal flora is different from that of normal rats; (2) RAGs can effectively regulate the structure of the intestinal flora in rats with CIRI and inhibit the disorder of the intestinal flora caused by cerebral ischaemia injury; (3) RAGs need to be metabolized into anthraquinone aglycones by the intestinal flora so that they can absorb into the blood; and (4) the changes in the intestinal flora caused by cerebral ischaemia injury can interfere with the absorption of anthraquinones compounds.

The intestine is the largest area of interaction between humans and the outside world. The total number of genes encoded by the different microorganisms in the intestine is 150–300 times higher than that of the whole human genome, so it is also called the “second genome” of humans [29]. An increasing number of studies have shown that the intestinal flora are closely related to the occurrence and development of central nervous system (CNS) diseases, and the CNS is closely related to the gastrointestinal tract [30, 31]. Recently, intestinal flora have been identified as an important participant in post-stroke pathophysiological events [32].

At present, there are many reports from around the world about the improvement of intestinal flora in the treatment of ischaemic cerebral injury. A previous study [33] confirmed that severe cerebral infarction can change the distribution of the intestinal flora, and faecal transplantation after cerebral infarction can significantly improve the prognosis of stroke. In our study, the results showed that the diversity and abundance of the intestinal flora in the model group were significantly lower than those in the normal group, which was consistent with the previous studies.

Rhubarb, as a TCM, is widely used into treat ischaemic diseases. Zhang et al. [34] studied the neuroprotective effect of emodin glycosides in vitro and in vivo and found that emodin-8-O-β-d-glucoside has a protective effect on the nerve injury induced by cerebral ischaemia–reperfusion. Our previous experiments also showed that RAGs can effectively reduce the cerebral infarction ratio in rats with CIRI. Audrey et al. [35] showed that rhubarb extract could improve the stability of the intestinal environment and had a certain impact on the composition of the intestinal flora. Our experimental results also showed that the abundance of the intestinal flora in rats with CIRI was significantly increased after treatment with RAGs. The structure of the intestinal flora of the rats in the drug group was more similar to that of the normal group. This is one of the bases of our speculation that the mechanism of RAGs’ anti-cerebral ischaemia effect is related to the intestinal flora.

The most common method of TCM administration is oral administration, and anthraquinone glycosides are compounds that are not easily absorb following oral administration. For many years, the absorption process of TCM, including anthraquinone glycosides, which are diffcult to absorb, has not been thoroughly studied and is controversial. Although many research results provide hints, this problem is not completely solved [36].

At present, studies have shown that most of these difficult-to-absorb TCMs can be hydrolysed into aglycones in the intestinal tract by the intestinal flora and enzymes. Aglycones can be partially absorb or further biotrans formed by intestinal flora. Yuan et al. [37] found that isoquercetin generated quercetin in the human body through o-deglycosylation metabolism of intestinal flora, and its antibacterial activity was stronger than that of its parent compound. In addition, the metabolism of glycosides by the intestinal flora mainly depends on the glycosidases that regulate the metabolism of glycosides in vivo, including β-glucosidase, β-xylanase and α-mannosidase, which are involved in the hydrolysis of various glycosidic bonds [38]. Studies have shown that glycosidase is dependent on a number of probiotics, which can mediate intestinal glycosylation metabolism [39]. Swann et al. [40] found that bifidobacteria can produce many glycosidases, such as β-glucosidase, β-fructofuranosidase, and d-xylanase.

In addition, TCM can induce changes in the structure of the intestinal flora. Xu et al. [41] isolated a heteropolysaccharide L2 from the *Lentinus edodes* fruiting body. L2 reduced the richness, diversity and evenness of the caecum and colon microbial community and remodelled the structure of the intestinal microflora. Does this observation mean that there is an interaction between the intestinal flora and the effective components of TCM? There are few studies on this interaction, which has also attracted the attention of scholars all over the world and has become a topic of international concern [9]. At present, the research on the metabolic effect of the intestinal flora on the active ingredients of TCM is mainly conducted from the following four aspects: 1. qualitative and quantitative analyses of metabolites; 2. determination of a specific metabolic strain that plays a role in the metabolism of an active ingredient of TCM; 3. analysis of the metabolites in intestinal contents, faeces, blood samples and urine samples; 4. comparison of the pharmacological activities of the original compounds and their metabolites after the effect of intestinal flora. However, our experiment focuses on the interaction between the intestinal flora and the effective components of TCM, and we should focus on the changes in the intestinal flora before and after administration and the changes in the effective components under the influence of the intestinal flora. Therefore, we chose to study the changes in the intestinal flora before and after the administration of RAGs and the metabolism of RAGs in vitro and in vivo to explore the interaction between the intestinal flora and the effective components of TCM. In the experiment, we found that RAGs were metabolized as free anthraquinone and gradually decomposed into other substances. The efficiency of metabolism and absorption of RAGs in normal rats was significantly higher than that in model rats. In addition, the relative abundance of *Lachnospiraceae* and *Prevotellaceae* decreased significantly, and the balance between the dominant bacteria in the rats with CIRI was improved after the administration of RAGs. Combined with the above, we speculate that there is an interaction between RAGs and intestinal flora. The intestinal flora is conducive to the metabolism and absorption of RAGs. In addition, RAGs can improve the structure of intestinal flora to promote the secretion of glycosidase and promote the metabolism and absorption of RAGs in the intestine, thus forming a positive cycle and promoting each other.

In conclusion, the results of this study suggest that CIRI leads to intestinal flora disorder in rats by decreasing the abundance and diversity of the intestinal flora. After the administration of RAGs, anthraquinone glycosides can be metabolized by intestinal flora and transformed into corresponding anthraquinone glycosides, which are absorb into the blood and improve the stability of the intestinal flora. The study of the relationship between the intestinal flora and medicine provides a basis for the theory of the brain-gut axis in the treatment of ischaemic brain diseases. In addition, the study of the interaction between glycosides and the intestinal flora can provide a reference for the action mechanism study of TCMs that are difficult to absorb. However, the current research still has some limitations. We have not verified the changes in the corresponding indicators or the characteristics of the rats with cerebral ischaemia after the intestinal flora was changed, and further studies will focus on those changes.

## Conclusions

In summary, our study suggests that the abundance and diversity of the intestinal flora in rats could decrease after cerebral ischaemia injury, thus leading to intestinal flora disorder. In addition, RAGs can effectively improve the intestinal flora abundance in rats with cerebral ischaemia injury. Moreover, RAGs need to be metabolized into their corresponding anthraquinone aglycones by intestinal flora so that they can be absorbed into the blood.

## Data Availability

The datasets used during the current study are available from the corresponding author on reasonable request.

## Abbreviations

RAGs: Rhubarb anthraquinone glycosides
SD: Sprague Dawley
MCAO: Middle cerebral artery occlusion
CIRI: Cerebral ischaemia–reperfusion injury
CMC-Na: Carboxymethylcellulose sodium
HPLC-UV: Ultraviolet high-performance liquid chromatography
CCA: Common carotid artery
ECA: External carotid artery
ICA: Internal carotid artery
TCM: Traditional Chinese medicine
PCA: Principal component analysis
CNS: Central nervous system
